# Frequency, causes, and duration of drug shortages in South Korea, 2018–2024: a nationwide descriptive analysis of regulatory reporting data and temporal patterns across the COVID-19 pandemic

**DOI:** 10.3389/fphar.2026.1931826

**Published:** 2026-09-28

**Authors:** Ji Young Kim, Eun Young Shin, Ju Won Park, Seung Soo Lee, Yoo Jin Lee, Yun Jae Jung, Min Ji Kim, Yu Kyoung Song, Chae Yun Kim, Se Hee Lee, Jong Hyuk Lee

**Affiliations:** 1 College of Pharmacy, Chung-Ang University, Seoul, Republic of Korea; 2 College of Pharmacy, Sookmyung Women’s University, Seoul, Republic of Korea

**Keywords:** active pharmaceutical ingredients, COVID-19, drug shortage, essential medicines, health policy, medicine supply, pharmaceutical supply chain

## Abstract

**Background:**

Drug shortages threaten treatment continuity and health-system resilience, yet empirical evidence from South Korea remains limited.

**Methods:**

We conducted a nationwide, retrospective descriptive analysis of 868 drug products (1,054 shortage reports) officially reported to the Ministry of Food and Drug Safety (MFDS) between January 2018 and February 2024. Shortages were characterized by frequency, reported cause (six categories), and duration, and compared between reportable and non-reportable drugs. Duration analyses were restricted to 360 temporary supply disruptions. To examine COVID-19-related temporal patterns, temporary supply disruptions during 2018–2023 were grouped into pre-COVID-19 (2018–2019), COVID-19 (2020–2021), and later COVID-19 (2022–2023) periods, defined a priori around the WHO pandemic declaration. Continuous variables were summarized as median (interquartile range, IQR) and categorical variables as counts (%). Analyses were descriptive; no causal inference was intended.

**Results:**

A directly substitutable product with the same active ingredient, strength, and dosage form was available for only 269 of 868 drugs (31.0%). Cause structures differed by event type: internal supply issues predominated among temporary supply disruptions (183/396 cause attributions, 46.2%) and business decisions among permanent discontinuations (418/720, 58.1%). Among temporary supply disruptions, regulatory constraints showed the longest durations (median 104 days; IQR 63.3–328) and the largest share lasting ≥366 days (25.0%). Products with ≥2 reported drug shortages were more frequent among orphan drugs (23.3%) and withdrawal-prevention drugs (21.1%), whereas Korean essential drugs showed a widely dispersed duration distribution (median 48 days; IQR 29.8–233.8; 25.6% > 180 days). The numbers of temporary supply disruptions were 53, 106, and 178 in the pre-COVID-19, COVID-19, and later COVID-19/recovery periods, respectively. While internal supply issues remained the leading reported cause throughout (69.8%, 53.4%, and 39.5%), the relative contribution of raw-material supply disruptions (11.3%–26.7%) and increased demand (9.4%–23.1%) grew.

**Conclusion:**

The patterns observed are consistent with structural vulnerabilities related to market incentives, regulatory processes, and API import dependence, with raw-material and demand-related causes becoming more prominent in the later study periods. Potential policy responses, each requiring separate evaluation, include supply diversification, domestic API production, cost-compensation for economically vulnerable essential medicines, and a risk-based early-warning system with severity-tiered responses.

## 1 Introduction

Medicines are essential public resources that must be available in a timely manner for the treatment and prevention of disease. Drug shortages—here used as an umbrella term for both temporary supply disruptions and permanent supply discontinuations of medicines that were previously marketed—pose substantial risks to health systems by undermining clinical care, straining budgets, and eroding public trust (Shukar et al., 2021). The coronavirus disease 2019 (COVID-19) pandemic, which triggered simultaneous surges in demand and widespread supply-chain disruption, further exposed shortages as a critical global challenge (U.S. Food and Drug Administration, 2019).

Shortages arise from complex, multifactorial mechanisms that are commonly grouped into supply- and demand-side drivers. Supply-side drivers include manufacturing disruptions, raw-material shortages, and logistical constraints, whereas demand-side drivers include just-in-time inventory practices, marketing dynamics, and tendering systems; these interact with the regulatory frameworks and market structures that govern pharmaceutical production and distribution (Shukar et al., 2021). Industry consolidation has amplified these vulnerabilities: according to the United States Food and Drug Administration (FDA), mergers and acquisitions have concentrated manufacturing in fewer sites, so that localised disruptions—plant closures, raw-material shortages, or transportation delays—can cascade into widespread shortages (U.S. Food and Drug Administration, 2019; World Health Organization, 2016).

Because shortages differ across health systems, regulatory regimes, and market structures, their drivers and appropriate remedies are context-specific (Shukar et al., 2021). Nonetheless, shortages have become increasingly prevalent worldwide (Huys and Simoens, 2013; Kweder and Dill, 2013; The Lancet, 2010), prompting extensive research on their causes, frequency, and consequences (U.S. Food and Drug Administration, 2019; European Cooperation in Science and Technology (COST), 2018; Ventola, 2011) and a range of policy responses, including France’s Dossier Pharmaceutique, Canada’s Tiered Notification and Communication Framework, and the United States FDA Drug Shortage Program (Ordre national des pharmaciens, 2024; Multi-Stakeholder Steering Committee on Drug Shortages, 2017; U.S. Food and Drug Administration, 2021). At the European level, coordinated action has developed rapidly, from a harmonised definition and notification framework to a Union list of critical medicines and legislative proposals intended to strengthen security of supply (European Commission, 2023a; European Medicines Agency et al., 2026; European Commission, 2025); a recent audit nonetheless concluded that structural problems remain, noting that national competent authorities reported 136 critical shortages to the European Medicines Agency between 2022 and October 2024 (European Court of Auditors, 2025). The reach of these programmes varies: in the United States, manufacturers are required to notify the FDA of manufacturing interruptions that could disrupt supply, yet this requirement is only weakly enforced and relies substantially on voluntary reporting (U.S. Government Accountability Office, 2025), and roughly 14% of prescription-drug shortages between 2017 and 2022 persisted for 6 months or longer (Callaway Kim et al., 2024).

South Korea offers an instructive but under-studied case. Its pharmaceutical market is dominated by generic products and depends heavily on imported APIs, and its regulatory and reimbursement arrangements differ from those of Western health systems (Lee et al., 2024; Yu and Lee, 2023; Um et al., 2022). Manufacturers, importers, and distributors are required to report anticipated production, importation, or supply discontinuations of designated shortage-relevant drugs to the Ministry of Food and Drug Safety (MFDS) (Ministry of Food and Drug Safety, 2026); nevertheless, supply-related shortages have continued to rise. Prior Korean work has examined community-pharmacy experiences (Lee et al., 2024) and price-adjustment negotiations (Yu and Lee, 2023), and our own group has developed machine-learning models to predict shortage duration and cause using the same national reporting system (Roe et al., 2025). What remains lacking is a comprehensive, population-level description of how often shortages occur, why they are reported to occur, how long they last, how these features differ between policy-protected (reportable) and other (non-reportable) medicines, and how they differed across the COVID-19 pandemic.

Against this background, this study provides a nationwide descriptive analysis of drug shortages reported in South Korea between 2018 and 2024, characterizing their frequency, reported causes, and duration, and comparing patterns across the pre-COVID-19, COVID-19, and later COVID-19 periods. In contrast to our previous study, which focused on predictive modelling (Roe et al., 2025), the present analysis is explicitly descriptive and policy-oriented, aiming to map the structural features of shortages and to inform national policy as well as other countries with comparable, generic-intensive and import-dependent pharmaceutical markets.

## 2 Materials and methods

### 2.1 Study design, data source, and study period

This was a retrospective, nationwide descriptive study based on MFDS reports of temporary supply disruptions and permanent supply discontinuations published through the national drug-information portal (Ministry of Food and Drug Safety, 2026). We included all 868 drug products for which a shortage was officially reported between 1 January 2018 and 6 February 2024. The study population therefore comprises the complete set of products notified to the MFDS during this period, not a sample of all medicines marketed in Korea; products that experienced no notified shortage, and shortages that occurred without notification, are absent by construction.

The study period was defined by data availability and completeness: the MFDS reporting system for production, importation, or supply interruptions and discontinuations began to generate systematically compiled, electronically retrievable records from 2018, and 6 February 2024 was the date of data extraction.

Records were identified by searching the portal for the study period and transcribed into a predefined spreadsheet template. Each record was verified against the corresponding portal entry by at least two of four investigators, with discrepancies resolved by discussion and any remaining disagreement referred to the senior author. The compiled dataset was screened for duplicate reports (matching product, marketing company and reported onset date) and for internal date inconsistency (a normalization date preceding the onset date, or an onset date outside the study period); no duplicates were found, and one record with a normalization date preceding its onset date was excluded from the duration analyses.

A “drug product” was defined at the level of active ingredient, dosage form, and strength; where the same product experienced more than one distinct shortage during the study period, each report was treated as a separate event for the analyses of cause and duration. The MFDS portal does not assign an episode identifier and contains no field linking one report to another; no minimum interval between reports was therefore imposed, and reports matching on product, marketing company, and reported onset date would have been consolidated as duplicates. The final dataset comprised 868 affected drug products and 1,054 reports, including 361 temporary supply disruptions and 693 permanent supply discontinuations. Throughout the manuscript, “temporary supply disruption” and “permanent supply discontinuation” are used as distinct terms, and “drug shortages” is used only where a statement applies to both event types.

### 2.2 Classification of shortage drugs

Shortage drugs were characterized by medication type (over-the-counter [OTC] vs. ethical/prescription [ETC]), formulation, manufacturing site (imported vs. locally manufactured), National Health Insurance reimbursement status, and marketing-company size. Manufacturing site was taken from the MFDS marketing-authorization type (import or domestic manufacture) and therefore refers to the finished product, not to the origin of the active pharmaceutical ingredient, since a domestically manufactured product may still be produced from imported APIs. Accordingly, manufacturing-site classification was not used as a measure of API origin or API import dependence. The availability of a directly substitutable product was determined using the Korea Pharmaceutical Information Center database (Korea Pharmaceutical Information Center). A directly substitutable product was operationally defined as another marketed product with the same active ingredient, strength, and dosage form. This deliberately narrow definition was intended to identify like-for-like substitutes rather than all clinically usable therapeutic alternatives.

Drugs were further classified as reportable or non-reportable under MFDS regulations (Ministry of Food and Drug Safety, 2018; Ministry of Food and Drug Safety, 2025). This distinction is policy-relevant because reportable drugs are those for which manufacturers and importers must notify the authorities of anticipated supply problems; they represent the segment of the market that current Korean policy explicitly seeks to protect, and comparing them with non-reportable drugs indicates whether that safety net aligns with where shortages actually concentrate. Reportable drugs were subdivided into four categories (Supplementary Table S1): withdrawal-prevention drugs, orphan drugs, Korean essential drugs, and other reportable drugs. These designations were not mutually exclusive, such that a single drug product could belong to more than one category. Reportable products that did not meet any of the first three designations were classified as other reportable drugs. Withdrawal-prevention drugs are clinically important but economically unattractive medicines that are formally designated—and eligible for cost compensation—to prevent their withdrawal from the market; the term denotes prevention of market withdrawal and does not refer to medicines for managing substance withdrawal. Orphan drugs were defined as products formally designated under Korean regulation for rare diseases affecting ≤20,000 patients domestically or in circumstances in which appropriate alternative treatment is lacking. Korean essential drugs were defined as medicines formally designated by the Ministry of Health and Welfare and the MFDS as necessary for public-health purposes but potentially difficult to supply adequately through ordinary market mechanisms. Detailed regulatory definitions and classification criteria are provided in Supplementary Table S1.

### 2.3 Frequency, causes, and duration

We first examined the overall distribution of reported causes across all shortage reports. Because a single report could cite more than one cause, causes were tallied as attributions; the 1,054 shortage reports yielded 1,116 cause attributions. Of the 1,054 reports, 62 (5.9%) cited more than one cause. Causes were classified into six categories defined *a priori* from the free-text reasons recorded by the MFDS: increased demand, raw-material supply issues, regulatory constraints, internal supply issues, business decisions, and other or unknown. Operational definitions for all six categories are provided in Supplementary Table S2. Regulatory constraints, for example, comprise supply disruptions arising from license cancellation, suspension of manufacturing, importation or sales, product recalls, or customs-clearance delays. Cause classification was carried out by the same four investigators using overlapping assignments, so that each classification was independently reviewed by at least two investigators; discrepancies were resolved by discussion, with any unresolved case referred to the senior author. These categories were derived from the MFDS free-text entries rather than from an existing taxonomy, but correspond broadly to the root-cause groupings used in European work—manufacturing and quality problems, raw-material and supply-chain disruption, regulatory processes, demand-side factors, and economic or commercial decisions (European Commission, 2021; AESGP et al., 2019). Our scope is nonetheless wider than the harmonized European definition of a shortage, which covers situations in which supply does not meet demand at national level and explicitly excludes withdrawal of a marketing authorization (European Medicines Agency and Heads of Medicines Agencies, 2019), because we also include permanent discontinuations.

Frequency analyses used the full set of 1,054 reports, whereas duration analyses were restricted to temporary supply disruptions, for which onset and estimated resolution dates are available; permanent discontinuations were excluded from duration analyses because they have no resolution date. All 361 temporary supply disruptions carried a projected normalization date at the time of extraction, so none was excluded on the grounds of being unresolved, and 396 cause attributions were recorded among them. One record was excluded because its recorded normalization date preceded its recorded onset date, leaving 360 temporary supply disruptions (395 cause attributions) in the duration analyses. Shortage duration was defined as the interval from the reported onset of disruption to the MFDS-estimated date of supply normalization. This date is the normalization date projected by the reporting company and recorded by the MFDS, not a date on which restored supply was independently verified; durations are therefore reported rather than observed.

### 2.4 Assessment of temporal patterns across the COVID-19 pandemic

To examine whether shortage patterns differed across the course of the COVID-19 pandemic, temporary supply disruptions reported between 1 January 2018 and 30 December 2023 were assigned to three periods defined *a priori*: pre-COVID-19 (2018–2019), COVID-19 (2020–2021), and later COVID-19/recovery (2022–2023). The COVID-19 window begins in 2020, the year the World Health Organization declared a public-health emergency of international concern (January 2020) and characterized COVID-19 as a pandemic (March 2020), capturing the period of acute global supply-chain disruption; the later COVID-19/recovery window reflects the subsequent transition toward endemicity and the relaxation of pandemic-era restrictions. Partial-year 2024 data were excluded from this comparison to avoid bias from an incomplete year. For each period we described the number of temporary supply disruptions, the number persisting beyond 6 months, and the distribution of reported causes. Because the analysis is descriptive, we report changes in counts and proportions across periods and do not attribute these changes causally to the pandemic. Reporting requirements changed during the study period: Korean essential drugs were added to the list of products subject to mandatory notification in December 2022 (Ministry of Health and Welfare, 2022).

### 2.5 Statistical analysis

All analyses were descriptive. Categorical variables were summarized as frequencies and percentages, and shortage durations—which were right-skewed—as medians with interquartile ranges (IQRs); means, standard deviations, and ranges are additionally reported for duration. No formal hypothesis tests or inferential trend models were applied, consistent with the descriptive aim of the study and the reliance on aggregated administrative reports. Denominators are reported alongside percentages throughout the tables to make the basis of each proportion explicit. Missing values were not imputed. Records with missing information on a given variable were retained and reported as a separate “unknown” category rather than excluded, so that the denominator remains the full set of 868 drug products for every product-level proportion.

## 3 Results

### 3.1 Characteristics of shortage drugs

The characteristics of the 868 drugs affected by shortages are summarized in Table 1. Across these products, 1,054 shortage reports were identified, comprising 361 temporary supply disruptions (34.3%) and 693 permanent supply discontinuations (65.7%). Most affected products were prescription (ethical, ETC) medicines (762, 87.8%). Imported and locally manufactured products were represented almost equally (407 [46.9%] and 396 [45.6%], respectively; manufacturing site unknown for 65 [7.5%]). Most drugs were reimbursed under National Health Insurance (604, 69.6%), and the majority were marketed by medium-sized companies with annual sales of 100 billion to one trillion Korean Won (510, 58.8%), with 41 products (4.7%) delisted from reimbursement and company size unknown for 79 (9.1%). Directly substitutable products with the same active ingredient, strength, and dosage form were available for only 269 drugs (31.0%), while 507 (58.4%) had none and substitutability could not be determined for 92 (10.6%). Of the 868 drugs, 484 were reportable and 384 were non-reportable; within the reportable group (categories not mutually exclusive), 71 were withdrawal-prevention drugs, 30 orphan drugs, 132 Korean essential drugs, and 276 other reportable drugs.

**TABLE 1:** Characteristics of shortage drugs in South Korea, 2018–2024.

Variable	n	%
Reported drug shortages (N = 1,054)		
Temporary supply disruptions	361	34.3%
Permanent supply discontinuations	693	65.7%
Affected drug products (N = 868)		
Medication type		
Ethical (prescription)	762	87.8%
Over the counter	106	12.2%
Drug formulation		
Oral	424	48.8%
Parenteral	315	36.3%
Topical	129	14.9%
Manufacturing site		
Imported	407	46.9%
Locally manufactured	396	45.6%
Unknown	65	7.5%
Reimbursement status		
Reimbursed	604	69.6%
Non-reimbursed	223	25.7%
Delisted	41	4.7%
Company size (KRW, Korean won)		
>1 trillion KRW	117	13.5%
100 billion–1 trillion KRW	510	58.8%
<100 billion KRW	162	18.7%
Unknown	79	9.1%
Directly substitutable product		
Available	269	31.0%
Not available	507	58.4%
Unknown	92	10.6%
Drug category (N = 868)		
Reportable drugs	484	55.8%
Withdrawal-prevention drugs	71	14.7% (of 484)
Orphan drugs	30	6.2% (of 484)
Korean essential drugs	132	27.3% (of 484)
Other reportable drugs	276	57.0% (of 484)
Non-reportable drugs	384	44.2%

A directly substitutable product was defined as another marketed product with the same active ingredient, strength, and dosage form; “unknown” denotes products for which the presence of such a product could not be determined. “Delisted” denotes products removed from the National Health Insurance benefit list. Percentages for reportable-drug subcategories were calculated within the 484 reportable products; all other percentages use the full set of 868 drug products as the denominator. Reportable-drug subcategories are not mutually exclusive; therefore, a drug product may be included in more than one subcategory, and percentages across these subcategories do not sum to 100%. Other reportable drugs were defined as reportable products that did not belong to any of the three designated categories above. KRW, Korean Won.

### 3.2 Causes and frequency of shortages

The 1,054 reports yielded 1,116 cause attributions, and the two event types showed different cause structures (Table 2). Internal supply issues predominated among temporary supply disruptions (183/396, 46.2%), whereas business decisions predominated among permanent supply discontinuations (418/720, 58.1%) and accounted for only 11 attributions (2.8%) among temporary disruptions. Commercially driven supply problems therefore manifested overwhelmingly as permanent withdrawal from the market rather than as temporary interruption of supply. Cause distributions by drug category are presented in Table 2, and recurrence and duration by drug category are summarized in Table 3. Recurrent events (≥2 reports for the same product) were more common among orphan drugs (23.3%) and withdrawal-prevention drugs (21.1%) than among other reportable subgroups, while most non-reportable drugs were the subject of a single report (single-report rate 92.2%).

**TABLE 2:** Reported causes by drug category and event type.

Cause of shortage	Withdrawal-prevention	Orphan	Korean essential	Other reportable	Non-reportable	Total
Temporary supply disruptions	55	24	96	193	64	396
Increased demand	18 (32.7)	5 (20.8)	27 (28.1)	34 (17.6)	11 (17.2)	80 (20.2)
Raw-material supply issues	20 (36.4)	–	25 (26.0)	39 (20.2)	9 (14.1)	82 (20.7)
Regulatory constraints	2 (3.6)	5 (20.8)	6 (6.2)	12 (6.2)	11 (17.2)	32 (8.1)
Internal supply issues	11 (20.0)	14 (58.3)	35 (36.5)	100 (51.8)	29 (45.3)	183 (46.2)
Business decisions	2 (3.6)	–	1 (1.0)	5 (2.6)	3 (4.7)	11 (2.8)
Other or unknown	2 (3.6)	–	2 (2.1)	3 (1.6)	1 (1.6)	8 (2.0)
Permanent supply discontinuations	51	18	107	176	382	720
Increased demand	2 (3.9)	–	1 (0.9)	–	1 (0.3)	3 (0.4)
Raw-material supply issues	11 (21.6)	–	14 (13.1)	38 (21.6)	40 (10.5)	102 (14.2)
Regulatory constraints	3 (5.9)	2 (11.1)	11 (10.3)	11 (6.2)	54 (14.1)	81 (11.2)
Internal supply issues	7 (13.7)	3 (16.7)	13 (12.1)	23 (13.1)	43 (11.3)	89 (12.4)
Business decisions	28 (54.9)	13 (72.2)	61 (57.0)	97 (55.1)	231 (60.5)	418 (58.1)
Other or unknown	–	–	7 (6.5)	7 (4.0)	13 (3.4)	27 (3.8)

Values are the number of cause attributions, n (%), with percentages calculated within each drug category. A single report could cite more than one cause, so cause attributions exceed the number of reports: the 361 temporary supply disruptions yielded 396 cause attributions and the 693 permanent supply discontinuations yielded 720. Drug categories are not mutually exclusive, so a product may contribute to more than one column and column totals therefore exceed the overall total. Percentages may not sum to 100 due to rounding. Reports citing substitution by another product as the reason were included in the “other or unknown” category.

**TABLE 3:** Recurrence and duration by drug category.

Drug category	≥2 reported drug shortages, % (n/N)	Duration of temporary supply disruptions ≤90 days, n (%)	91–180 days, n (%)	>180 days, n (%)	Median days (IQR)
Withdrawal-prevention drugs (n = 45)	21.1 (15/71)	30 (66.7)	6 (13.3)	9 (20.0)	56 (27.0–122.0)
Orphan drugs (n = 23)	23.3 (7/30)	17 (73.9)	3 (13.0)	3 (13.0)	35 (20.0–93.0)
Korean essential drugs (n = 82)	18.9 (25/132)	50 (61.0)	11 (13.4)	21 (25.6)	48 (29.8–233.8)
Other reportable drugs (n = 177)	19.9 (55/276)	115 (65.0)	40 (22.6)	22 (12.4)	62 (27.0–121.5)
Non-reportable drugs (n = 61)	7.8 (30/384)	37 (60.7)	7 (11.5)	17 (27.9)	71 (26.0–187.5)

Recurrence was assessed at the product level as the presence of ≥2 reported drug shortages of either type during the study period, with the number of affected products in that category as the denominator. Duration statistics are restricted to temporary supply disruptions, and the number of disruptions contributing to each row is given in the first column. Reportable categories are not mutually exclusive, so a product may be included in more than one row. Durations were calculated from the reported onset date to the MFDS-estimated normalisation date and are therefore reported rather than observed durations. Cause distributions by drug category are given in Table 2. IQR, interquartile range.

### 3.3 Duration of temporary supply disruptions by drug category and reported cause

Most temporary supply disruptions across all categories had a reported duration within 90 days (Table 3). Orphan drugs had the highest proportion of temporary supply disruptions resolved within 90 days (73.9%), followed by withdrawal-prevention drugs (66.7%), other reportable drugs (65.0%), Korean essential drugs (61.0%), and non-reportable drugs (60.7%). Non-reportable drugs showed the highest proportion of prolonged disruptions lasting >180 days (27.9%), followed by Korean essential drugs (25.6%) and withdrawal-prevention drugs (20.0%). In contrast, orphan drugs and other reportable drugs had lower proportions of disruptions lasting >180 days (13.0% and 12.4%, respectively). Median duration was shortest for orphan drugs (35 days, IQR 20.0–93.0) and longest for non-reportable drugs (71 days, IQR 26.0–187.5), while Korean essential drugs showed substantial variability in duration (median 48 days, IQR 29.8–233.8).

Duration also differed by reported cause (Table 4). Temporary supply disruptions attributed to increased demand most often resolved within 90 days (60/80, 75.0%; median 40 days, IQR 20–81.5), as did those attributed to internal supply issues (122/183, 66.7%; median 55 days, IQR 24–118) and to raw-material supply issues (49/82, 59.8%; median 67 days, IQR 35–151). Disruptions attributed to regulatory constraints showed the longest central tendency and the widest dispersion (median 104 days, IQR 63.3–328) and accounted for the largest share lasting ≥366 days (8/32, 25.0%).

**TABLE 4:** Duration of temporary supply disruptions by reported cause.

Duration	Increased demand (n = 80)	Raw-material supply issues (n = 82)	Regulatory constraints (n = 32)	Internal supply issues (n = 183)	Business decisions (n = 11)	Other or unknown (n = 7)
0–90 days	60 (75.0%)	49 (59.8%)	15 (46.9%)	122 (66.7%)	6 (54.5%)	6 (85.7%)
91–180 days	9 (11.3%)	16 (19.5%)	3 (9.4%)	35 (19.1%)	2 (18.2%)	—
181–270 days	—	4 (4.9%)	6 (18.7%)	6 (3.3%)	—	—
271–365 days	7 (8.7%)	10 (12.2%)	—	5 (2.7%)	3 (27.3%)	—
≥366 days	4 (5.0%)	3 (3.7%)	8 (25.0%)	15 (8.2%)	—	1 (14.3%)
Median (IQR)	40 (20–81.5)	67 (35–151)	104 (63.3–328)	55 (24–118)	38 (30–249.3)	35 (27.5–48)
Mean	88	118	226	111	119	90

Durations were calculated from the reported onset date to the MFDS-estimated normalisation date. Of the 361 temporary supply disruptions (396 cause attributions), one report with an invalid date sequence was excluded from the duration analysis, leaving 360 disruptions and 395 cause attributions. IQR, interquartile range.

### 3.4 Temporal patterns in shortages across the COVID-19 pandemic

Between 1 January 2018 and 30 December 2023, 337 temporary supply disruptions were reported. The observed number of temporary supply disruptions was higher in the later study periods: 53 in the pre-COVID-19 period (2018–2019), 106 in the COVID-19 period (2020–2021), and 178 in the later COVID-19 period (2022–2023). Of all temporary supply disruptions, 52 persisted beyond 6 months, and the number of such prolonged events also rose over time (Figure 1).

**FIGURE 1:**
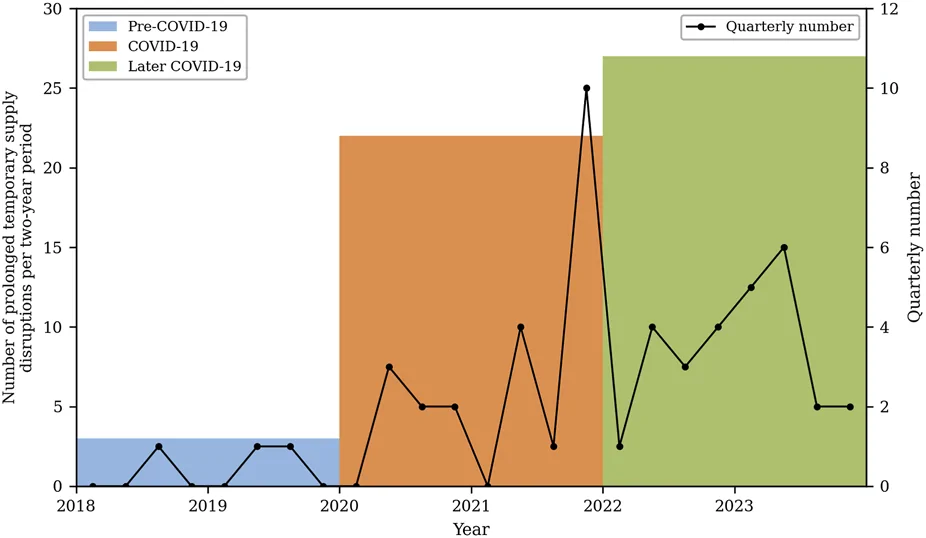
Distribution of prolonged temporary supply disruptions across the pre-COVID-19, COVID-19, and later COVID-19 periods (2018–2023). Bars show the total number of temporary supply disruptions lasting >6 months within each 2-year period (pre-COVID-19, 2018–2019, n = 3; COVID-19, 2020–2021, n = 22; later COVID-19, 2022–2023, n = 27). The black line shows the corresponding quarterly counts. In total, 52 temporary supply disruptions lasted >6 months during 2018–2023. Partial-year 2024 data were excluded from the COVID-19 periods comparison.

The distribution of reported causes differed in parallel (Figure 2). In the pre-COVID-19 period, internal supply issues accounted for most cause attributions (69.8%). This remained the leading category in the COVID-19 (53.4%) and later COVID-19 (39.5%) periods, but its relative share fell as other causes grew: raw-material supply issues rose from 11.3% (pre-COVID-19) to 13.8% (COVID-19) and 26.7% (later COVID-19), and increased demand rose from 9.4% to 12.1% and 23.1% over the same periods. Thus, although internal supply issues consistently represented the largest single share of temporary supply disruptions, the relative contribution of raw-material and demand-related factors increased across the study periods.

**FIGURE 2:**
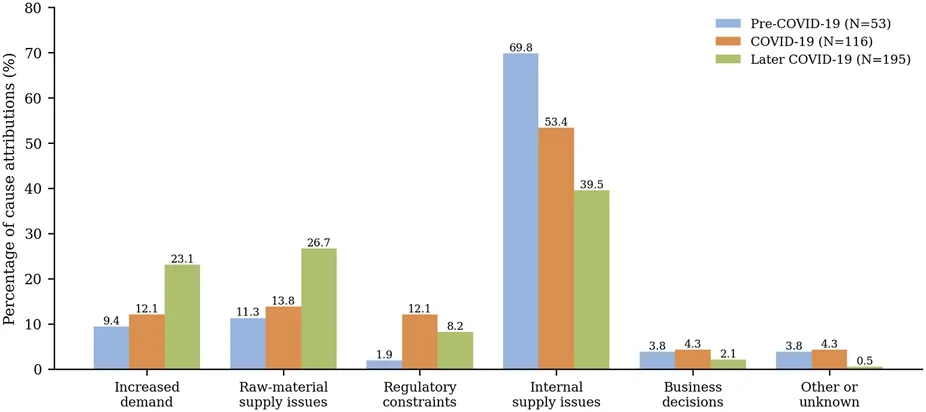
Distribution of reported causes of temporary supply disruptions across the pre-COVID-19, COVID-19, and later COVID-19 periods. Percentages were calculated from the total number of cause attributions recorded within each period: pre-COVID-19 (2018–2019), N = 53; COVID-19 (2020–2021), N = 116; and later COVID-19 (2022–2023), N = 195. A single temporary supply disruption report could contribute more than one cause attribution; therefore, these denominators represent cause attributions rather than unique disruption reports. Partial-year 2024 data were excluded.

## 4 Discussion

In this nationwide analysis of all drug shortages reported to the MFDS between 2018 and 2024, four findings stand out. First, only about one-third of affected products had a directly substitutable product meeting our predefined criteria, underscoring the clinical fragility created by shortages. Second, business decisions were the most frequently reported cause of permanent supply discontinuations, whereas the longest durations among temporary supply disruptions were associated with regulatory constraints. Third, descriptive patterns varied across policy-designated drug groups: orphan drugs and withdrawal-prevention drugs had relatively high proportions of products with repeated reported drug shortages, while Korean essential drugs showed the widest variation in duration, with a quarter of temporary supply disruptions lasting more than 180 days. These subgroup findings should be interpreted cautiously given the small numbers in some categories, particularly orphan drugs. Fourth, the composition of reported cause attributions differed across the predefined COVID-19-related calendar periods, with raw-material supply and increased-demand attributions accounting for larger proportions in the later COVID-19 period, while internal supply issues remained the largest single reported category. These temporal patterns are descriptive and do not establish a causal effect of COVID-19.

Only 269 of 868 affected products (31.0%) had another marketed product that met our narrow definition of direct substitutability—namely, the same active ingredient, strength, and dosage form—while for a further 92 products (10.6%) substitutability could not be determined. This finding indicates limited availability of like-for-like substitutes but should not be interpreted as showing that no clinically usable alternative existed for the remaining products, because medicines with different strengths, dosage forms, or active ingredients may be appropriate depending on the indication, patient characteristics, and clinical judgement. Previous research has suggested that substitution necessitated by shortages may increase medication-management complexity and the potential for medication errors (Kim and Kwon, 2013); however, the present study did not measure prescribing changes, medication errors, treatment outcomes, or other patient-safety outcomes. Accordingly, these potential consequences provide context for the findings rather than outcomes demonstrated by this study. Standardized and regularly updated information on potential substitution options could complement shortage notifications and support clinical decision-making.

That business decisions dominated the causes reported for permanent supply discontinuations—encompassing mergers, contractual changes, prioritization of new launches, and low profitability—illustrates the limits of relying on market mechanisms alone to guarantee supply and is consistent with international evidence that economically unattractive products are disproportionately vulnerable (USP, 2024a). A permanent discontinuation translates into clinical unavailability only where a substitutable product is absent; because a directly substitutable product was available for only 31.0% of affected products, commercially driven withdrawals in this dataset frequently left no like-for-like alternative on the market. At the same time, regulatory constraints, though less frequent, were associated with the longest and most dispersed durations, reflecting the time required to resolve licensing, recall, and compliance issues. Similar patterns of lengthening durations have been reported in the United States (USP, 2024a; USP, 2024b). The contrast between business-related causes, which arose almost entirely in the context of permanent withdrawal, and regulatory causes, which were infrequent but associated with the longest temporary disruptions, suggests that a single policy lever is insufficient: preventing the former calls for economic incentives, whereas mitigating the latter calls for regulatory agility and redundancy.

The distinctive behavior of policy-prioritized medicines deserves emphasis. Korean essential drugs showed the widest dispersion in duration, with a substantial minority persisting well beyond 6 months and the longest exceeding 1 year, while orphan and withdrawal-prevention drugs recurred most often. Many of these products share low demand, limited profitability, or a lack of alternatives, which makes them structurally prone to instability. Although recent Korean reforms require earlier notification of anticipated disruptions (Ministry of Food and Drug Safety, 2025), notification alone does not resolve the underlying economics. Additional measures—cost-compensation mechanisms for low-margin essential medicines, incentives for domestic production, and a post-crisis evaluation framework that classifies shortage severity in a tiered manner, as used elsewhere (Videau et al., 2019)—may help to match policy effort to where shortages actually concentrate. Comparable instruments are being developed in the European Union: the Union list of critical medicines selects products on the basis of the seriousness of the condition treated and the availability of appropriate alternatives (European Medicines Agency et al., 2026), and the proposed revision of the pharmaceutical legislation would require marketing authorization holders to maintain shortage prevention plans and to notify permanent discontinuation twelve months in advance (European Commission, 2023b). The alignment with our findings is direct: the availability of a substitutable product, which determines whether a discontinuation becomes a clinical shortage, is used in Europe as a criterion for prioritization rather than only as an outcome to be described. Notably, prolonged temporary supply disruptions were not confined to the reportable segment: non-reportable drugs showed both the longest median duration (71 days) and the highest proportion of disruptions lasting more than 180 days (27.9%), even though business decisions dominated their reported causes. The current reporting-based safety net therefore covers the products that policy has prioritized, but products outside it are not systematically less affected once a disruption has been reported.

The number of temporary supply disruptions was higher in each successive period, and the relative contribution of raw-material and demand-related causes grew, a pattern consistent with observations reported in the United States, China, and low- and middle-income countries (U.S. Food and Drug Administration, 2021; Sabogal De La Pava and Tucker, 2022; Song et al., 2023). Our dataset does not record API sourcing—the manufacturing-site variable distinguishes imported from domestically manufactured finished products, not their ingredient origin—so the following figures are drawn from separate national statistics. Whether this represents a lasting structural change or a transient shock cannot be determined from a descriptive analysis of this kind. South Korea depends heavily on imported APIs: the MFDS reported an API self-sufficiency rate of only 11.9% in 2022, rising to 25.6% in 2023 (Ministry of Food and Drug Safety, 2022), and China, India, and Japan together supplied more than 60% of imported APIs as of 2022 (Ministry of Food and Drug Safety, 2023). Heavy outsourcing to a few source countries increases exposure to export restrictions, geopolitical tensions, and logistical shocks (USP, 2024a; Socal et al., 2021), as seen when API-producing countries restricted exports during the pandemic (Chowdhury et al., 2021). As the United States Food and Drug Administration has noted, supply systems that rely heavily on outsourcing pose direct risks to patient safety and national health security when disruptions occur (U.S. Food and Drug Administration, 2019). These considerations point to national strategies that strengthen domestic API capacity, diversify import sources, foster regional cooperation, and integrate supply-security considerations into pricing and reimbursement—areas where market forces alone are unlikely to suffice. The share attributed to increased demand was largest in the later COVID-19 period (23.1%) rather than during 2020–2021 (12.1%). Two factors may contribute to this pattern: the MFDS system is oriented towards supply-side notification, so demand surges managed through allocation may not generate a formal report, and the most prominent demand-driven shortages in Korea, involving antipyretics and cold preparations, occurred during the Omicron waves of late 2022. These data should therefore not be read as indicating that demand pressure was absent earlier in the pandemic.

Our results should be read together with, and are distinct from, our previous predictive study, which analyzed the same underlying set of 1,054 MFDS reports (Roe et al., 2025). Whereas that work built machine-learning models to forecast shortage duration and cause, the present study provides the descriptive epidemiology—frequency, cause distribution, duration, and COVID-19-related temporal patterns, disaggregated by regulatory status—that such models require as a foundation and that policymakers need to prioritize interventions. Read jointly, the two studies suggest a pathway from description to prediction to a risk-based, severity-tiered early-warning system embedded in the national monitoring infrastructure.

## 5 Limitations

This study has several limitations. First, it relied on officially reported data and therefore probably underestimates the true extent of drug shortages, particularly for non-reportable products. Because the notification obligation applies only to reportable products, this under-capture is not uniform across the groups we compare, and differences between reportable and non-reportable drugs should be interpreted with that asymmetry in mind. Our narrow definition of direct substitutability may also underestimate the availability of clinically usable alternatives. Second, some causes were recorded under broad or ambiguous categories, limiting the specificity of the reported causes and their interpretation; because a single event could cite multiple causes, cause attributions exceed the number of events. This constraint is not specific to Korea: the European Commission’s study on medicine shortages likewise found that root-cause analysis across member states is limited by the heterogeneity and incompleteness of national shortage reporting (European Commission, 2021). Because no minimum interval between reports was applied, recurrence may also be overstated where a single prolonged disruption generated more than one notification. Third, estimated durations are based on MFDS-projected normalization dates rather than confirmed dates of supply stabilization in clinical practice and therefore may not reflect the actual duration experienced by healthcare institutions; the difference may run in either direction, since supply may in some cases be restored earlier than projected. Fourth, the manufacturing site was unknown for 7.5% of products and substitutability could not be determined for a further 10.6%, which limits the precision of the corresponding comparisons.

Fifth, the COVID-19-period comparison is descriptive: the observed increases coincide with, but cannot be causally attributed to, COVID-19, and the a priori period boundaries are a simplification. The reporting system may also under-capture demand-driven shortages managed without formal notification. Sixth, longitudinal trends in reporting volume were likely influenced by regulatory changes during the study period. Most notably, the notification designating medicinal products subject to mandatory reporting of production, import, or supply suspension was revised with effect from December 2022 to include Korean essential drugs (Ministry of Health and Welfare, 2022), substantially expanding the baseline number of monitored medications from that point onwards. Coupled with more active government monitoring during the COVID-19 period, the observed increase in reporting volume may partly reflect an expanded reporting scope rather than an increase in shortage events alone. Nevertheless, while these systemic temporal patterns may confound the absolute volume of reports over time, they are less likely to account for the differences in cause composition between event types and drug categories that we report. Finally, the findings reflect South Korea’s specific pharmaceutical market, shortage-reporting system, and regulatory context and therefore may not generalize to other systems. Future work integrating manufacturer, wholesaler, and institutional data, and developing risk-based predictive models, would strengthen anticipation and management of shortages.

## 6 Conclusion

The patterns observed in South Korea are consistent with structural vulnerabilities related to market incentives, regulatory processes, and dependence on imported APIs, with raw-material and demand-related causes becoming more prominent in the later study periods. These findings identify where reported supply problems concentrate and therefore where policy attention may be warranted. Potential responses, each of which would require separate evaluation, include diversification of supply sources, support for domestic API production, cost-compensation for economically vulnerable essential medicines, and a risk-based early-warning system with severity-tiered response mechanisms. Because the patterns described here are shared by many generic-intensive, import-dependent markets, these findings offer a reference for broader international efforts to strengthen medicine-supply resilience.

## Data Availability

The original contributions presented in the study are included in the article/Supplementary Material, further inquiries can be directed to the corresponding authors.
