# Supplementary material for: Frequency, causes, and duration of drug shortages in South Korea, 2018–2024: a nationwide descriptive analysis of regulatory reporting data and temporal patterns across the COVID-19 pandemic
Source: Front Pharmacol. 2026 Sep 28;17:1931826. doi: 10.3389/fphar.2026.1931826 (PMC13655078; doi:10.3389/fphar.2026.1931826)
Supplement: Supplementary file 1 [file Table1.docx]

Supplementary Material

Frequency, causes, and duration of drug shortages in South Korea, 2018–2024: a nationwide descriptive analysis of regulatory reporting data and temporal patterns across the COVID-19 pandemic

*Ji Young Kim, Eun Young Shin, Ju Won Park, Seung Soo Lee, Yoo Jin Lee, Yun Jae Jung, Min Ji Kim, Yu Kyoung Song, Chae Yun Kim, Se Hee Lee and Jong Hyuk Lee*

# 1 Supplementary Table S1

**Supplementary Table S1.** Drug classification by regulatory status.

| **Category** | **Definition** |
| --- | --- |
| **Reportable drugs** | Medications subject to mandatory reporting to the MFDS in cases of production suspension, import disruption, or supply discontinuation. Subdivided into four categories (below). |
| Withdrawal-prevention drugs | Drugs designated to prevent market withdrawal of essential medicines that are clinically important but economically unattractive to manufacture or import. Eligible for cost compensation to ensure continuous supply and reduce the financial burden on the national health insurance system. |
| Orphan drugs | Drugs designated for the treatment of rare diseases (conditions affecting ≤20,000 patients domestically) or diseases for which no appropriate alternative therapy exists; also includes drugs with improved safety or efficacy for rare diseases, drugs under clinical development for rare conditions, or drugs with limited supply that could substantially affect patient care. |
| Korean essential drugs | Medications designated by the Ministry of Health and Welfare and the MFDS, in consultation with relevant central administrative agencies, as essential for public-health purposes (e.g., infectious-disease control or radiation protection) but difficult to supply stably through market mechanisms alone. |
| Other reportable drugs | Reportable drugs that do not meet the criteria for withdrawal-prevention drugs, orphan drugs, or Korean essential drugs but remain subject to mandatory reporting under MFDS regulations. |
| **Non-reportable drugs** | Medications not subject to mandatory reporting under MFDS guidelines for production, importation, or supply discontinuation. |

# 2 Supplementary Table S2

**Supplementary Table S2.** Definitions of the six categories of causes of drug shortages, classified from information reported to the MFDS.

| **Cause category** | **Definition** |
| --- | --- |
| **Increased demand** | Sudden surges in demand due to events such as pandemics (e.g., COVID-19), outbreaks, or public-health emergencies. |
| **Raw-material supply issues** | Disruptions related to active pharmaceutical ingredient (API) or other raw-material shortages, quality failures, delays, price increases, or termination of supply contracts. |
| **Regulatory constraints** | Supply disruptions resulting from license cancellations; regulatory suspension of manufacturing, importation, or sales; product recalls; or customs-clearance delays. |
| **Internal supply issues** | Internal operational problems, including limited production capacity, changes or loss of manufacturing facilities, aging or malfunctioning equipment, quality-assurance failures, defective products, import delays, or adverse-reaction reports. |
| **Business decisions** | Strategic or financial decisions such as mergers and acquisitions, contractual disputes, prioritisation of new product launches, low profitability, operational restructuring, or financial deterioration. |
| **Other or unknown causes** | Causes that could not be clearly classified into the above categories due to insufficient or ambiguous information. |
